# The use of cognitive ability measures as explanatory variables in regression analysis

**DOI:** 10.1186/2193-8997-1-4

**Published:** 2012-10-09

**Authors:** Brian Junker, Lynne Steuerle Schofield, Lowell J Taylor

**Affiliations:** 1 Dept. of Statistics, Carnegie Mellon University, 5000 Forbes Avenue, Pittsburgh, PA 15213; 2 Dept. Mathematics & Statistics, Swarthmore College, 500 College Avenue, Swarthmore, PA, 19086; 3 Heinz College, Carnegie Mellon University, 5000 Forbes Avenue, Pittsburgh, PA 15213

**Keywords:** Labor economics, Structural equations modeling, Item response theory

## Abstract

Cognitive ability measures are often taken as explanatory variables in regression analysis, e.g., as a factor affecting a market outcome such as an individual’s wage, or a decision such as an individual’s education acquisition. Cognitive ability is a latent construct; its true value is unobserved. Nonetheless, researchers often assume that a *test score*, constructed via standard psychometric practice from individuals’ responses to test items, can be safely used in regression analysis. We examine problems that can arise, and suggest that an alternative approach, a “mixed effects structural equations” (MESE) model, may be more appropriate in many circumstances.

## Background

Cognitive test scores—whether from standardized achievement tests or from cognitive items on surveys—are used widely as explanatory or control variables in the social sciences.^a^ Political scientists use cognitive test scores as a descriptive demographic variable to characterize voting behavior (Venezky and Kaplan 1998). Health researchers are interested in how cognitive ability (and other latent constructs, e.g., depression) affects a patient’s understanding of and likelihood of following prescribed therapies (Schillinger et al. 2002). Social scientists control for “ability” in analyses that seek to evaluate the role of parental financial resources in determining post-secondary education (Dynarski 2002).

The analyses in such studies often proceed using linear regression models, such as

(1) $$y_{i}=\beta_{0}+\beta_{1}\theta_{i}+\beta_{2}Z_{i}+\beta_{3}W_{i}+\varepsilon_{i},$$

where *y_i_* is an outcome for individual *i*, *θ_i_* is a measure of the latent construct from a test, *Z_i_* indicates the contrast of central interest, and *W_i_* represents other covariates. For example, equation (1) has been used extensively in labor economics—in analyses intended to tease apart the influences of cognitive ability (as measured by *θ_i_*) and possible market effects of race or gender status (*Z_i_*) on log wage (*y_i_*). Prominent examples include Neal and Johnson (1996), Bollinger (2003), and Lang and Manove (2011).

The variable *Z_i_* is typically a 0/1 indicator for two groups. In the case of designed experiments (or natural quasi-experiments), the groups are *treatment* vs. *control*; in the case of observational studies that examine disparate outcomes for some specific group (e.g., a racial or ethnic group), we might refer to groups as *focal* vs. *reference* (Holland and Thayer 1988; Penfield and Camilli 2007). In a study of wage disparities between reference and focal groups, for example, *β*_1_ is the “return to cognitive ability,” and *β*_2_ is intended to measure disparate treatment in the labor market for members of the focal group.

A key obstacle to obtaining (asymptotically) unbiased estimates of *β*_1_ and *β*_2_ in (1) is the possibility of measurement error in *θ_i_*. Indeed, standard theory for scoring cognitive tests (Lord and Novick 1968) takes as axiomatic that *θ_i_* is a latent variable, and any proxy for it entails some measurement error. It is well known that regression coefficients are biased if measurement error is ignored. Standard approaches for dealing with this sort of measurement error include the use of nonparametric bounds, instrumental variable estimation, and direct modeling.

Nonparametric bounds are invaluable, especially when little is known about the data-generating process. For example, Bollinger (2003) shows that failing to correct for measurement error in (1) can lead the researcher to estimate a black-white wage difference that is biased downward, and calculates Klepper and Leamer (1984) bounds for the regression coefficients. Such nonparametric bounds can be wide. In Bollinger’s empirical example, which studies the impact of race on log wage (the focal group is black and the reference group is white), the estimated bounds for men are (−0.07, 1.26) and for women are (0.04, 1.39).^b^

Instrumental variable methods provide a standard answer to measurement error in a regressor when instruments of sufficient quality and relevance can be found. However, the measurement error in cognitive test scores is itself exogenous; it is dependent only upon the measurement procedure in a well-designed test, and not on omitted variables that might be associated with any outcome of interest. Thus instrumental variables will typically not be of use in correcting for it. In cases where instruments can be found, one would expect them to be rather weak (leading to problems discussed in Staiger and Stock (1997)).

When *θ_i_* is to be obtained from a well-constructed cognitive assessment using standard modern technology (such as the assessments listed in footnote a), a direct model for measurement error has already been used as part of the quality-control process of constructing the test, and is available to produce scores *θ_i_* with known measurement error properties. The class of models used to construct many standard cognitive assessments is known as item response theory (IRT) models (van der Linden and Hambleton 1997).

The existence of IRT as a direct model obviates the need for refining nonparametric bounds or searching for suitable instruments to adjust for measurement error in cognitive test scores. Indeed, because the cognitive assessment was *constructed* to fit this model, answers obtained using this model have more authority than answers using other methods, regardless of raw comparisons of effect size estimates, statistical significance, etc. The class of IRT models is flexible enough that it should be considered as a direct model for measurement error even in cases in which number-correct score might be used, even if the test was not constructed using IRT techniques.

In this paper we explore the use of IRT as a direct model for measurement error in cognitive ability in applications common in labor economics. We consider both linear models such as (1) and generalized linear models such as logistic regression. First, we review the basic features of the IRT family of models, and their role in determining measurement error. Second, we combine the IRT model with linear and generalized linear models along the lines of equation (1). Third, we illustrate the methodology in two applications, one linear and one non-linear.

We note finally that our work is focused on a particular kind of error in a particular kind of variable in errors-in-variables regressions such as equation (1): measurement error inherent in psychometric measures of cognitive status. We have nothing to add here about problems that emerge due to other variables being measured with error, e.g., self-reported years of schooling, parents’ schooling, parents’ income, etc.

## Models used to construct cognitive test scores

Well-constructed cognitive tests use statistical methodology as a quality control device in the construction of the test. The process inevitably involves the interplay of defining and refining the construct to be measured, designing test items to measure it, defining a space of responses and scoring rules for each possible response in the space, and finally developing a statistical measurement model to assemble responses to test items into an observed “score” that measures the construct with some quantifiable level of error (e.g., Wilson 2005). Candidate test items that do not produce data consistent with the measurement model are rejected in favor of those that do; in this sense, the data from a well-built cognitive test fits the statistical measurement model *by construction*.

Some cognitive assessments—especially ones intended for smaller-scale use—are built using a measurement model called *classical true-score theory* (CTT). Under this model the observed test score *X_i_* is expressed in terms of a true score (or latent cognitive status) *θ_i_* and measurement error *ν_i_*,

(2) $$X_{i}=\theta_{i}+\nu_{i}.$$

This model, together with standard distributional assumptions, has been useful for thinking about measurement error *ν_i_* when *X_i_* is the total score (number-correct score). For example, standard psychometric formulae such as the Spearman-Brown formula for expressing the reliability of a total score as a function of test length, or Cronbach’s alpha lower-bound for reliability—are based on elaborations of CTT (see Lord and Novick 1968).

More commonly, especially for large-scale assessments or for cognitive portions of large-scale surveys, the measurement model used to build the test is an *item response theory* (IRT) model. IRT models can be thought of as a generalization of mixed effects logistic regression models (Stiratelli et al. 1984). Instead of modeling the total score *X_i_* for individual *i*, IRT models focus on the individual responses *X_ij_* of individual *i* to test item *j*.

One of the most common IRT models in cognitive testing is the *three-parameter logistic* (3PL) model. Letting *X_ij_* = 0 for an incorrect answer and *X_ij_* = 1 for a correct answer, the 3PL model posits the probability of a correct response as

(3) $$P_{j}(\theta_{i})\equiv P[X_{ij}=1]=c_{j}+\frac{1-c_{j}}{1+\exp[a_{j}(\theta_{i}-b_{j})]},$$

where *θ_i_* is the latent “amount” of cognitive skill for individual *i*, usually treated as a random effect, and *a_j_*, *b_j_*, and *c_j_* are parameters reflecting characteristics of the item, usually treated as fixed effects. The parameter *b_j_* is the “item difficulty”—the larger is *b_j_*, the lower is the probability that *X_ij_* = 1. The parameter *c_j_* is a “guessing parameter” measuring the likelihood that a very low-ability examinee would respond correctly simply by guessing. The parameter *a_j_* measures how influential changes in *θ_i_* are on changes in *P*[*X_ij_* = 1], and conversely, drives the level of measurement error. Sijtsma and Junker (2006) give a brief overview of CTT, IRT and related models; Rao and Sinharay (2007) provide more in-depth reviews.

### Measurement error in IRT models

Measurement error in IRT models can be thought of as the standard error of estimation for *θ_i_* from the pattern of scored item responses from examinee *i* (van der Linden and Hambleton 1997). For example, conditional on knowing the item parameters *a_j_*, *b_j_* and *c_j_*, the likelihood for a particular pattern of wrong and right answers on a test of *J* items following the 3PL model is

$$P[X_{i1}=x_{i1},\ldots ,X_{iJ}=x_{iJ}|\theta_{j}]=\prod_{j=1}^{J}P_{j}{\left(\theta_{i}\right)}^{x_{ij}}{\left(1-P_{j}(\theta_{i})\right)}^{1-x_{ij}},$$

from which it is straightforward to calculate that the Fisher information for estimating *θ_i_* is

(4) $$I(\theta_{i})=\sum_{j=1}^{J}\frac{{\left[P_{j}^{\prime }(\theta_{i})\right]}^{2}}{P_{j}(\theta_{i})(1-P_{j}(\theta_{i}))};$$

the measurement error is then $SE(\theta_{i})=1/\sqrt{I(\theta_{i})}$.

This immediately suggests that the IRT model imposes some constraints on the magnitude of the measurement error for *θ_i_*. Indeed, taking the simplest case, if all *a_j_* = 1 and *b_j_* = *c_j_* = 0, then

(5) $$SE(\theta_{i})=\left(\frac{1}{\sqrt{J}}\right)\;\left(\frac{1+e^{\theta_{i}}}{e^{\theta_{i}/2}}\right).$$

**Figure 1:**
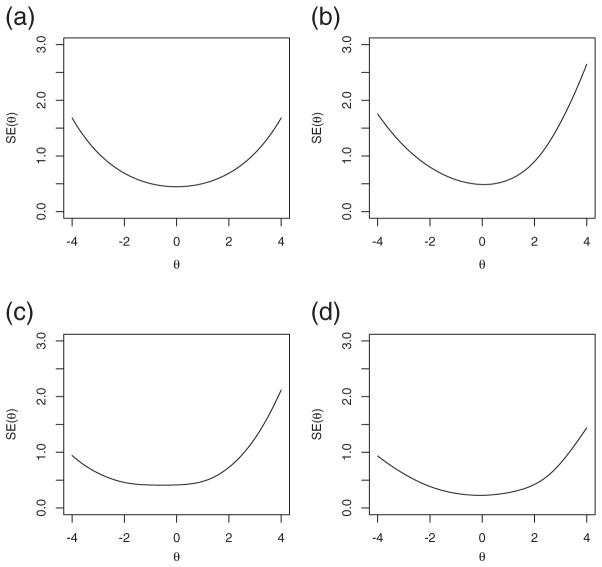
Measurement error under IRT models: (a) for the simple model whose measurement error is expressed in equation (5); (b), (c), (d) for more typical 3PL tests of length 20, 40 and 80 items **(a)** Measurement error for simple model, J = 20, **(b)** Measurement error for typical 3PL, J = 20, **(c)** Measurement error for typical 3PL, J = 40, **(d)** Measurement error for typical 3PL, J = 80.

While in principle *θ_i_* may be any real number, as a practical matter any *θ_i_* less than roughly −4 is indistinguishable in data from any other; and similarly for any *θ_i_* greater than roughly +4. The first panel of Figure 1 depicts the measurement error curve for equation (5) over the range −4 ≤ *θ* ≤ 4, for a test of length *J* = 20 items; the other three panels illustrate the measurement error for more typical 3PL tests. (See Schofield (2008) for additional discussion.)

Such a precise specification of measurement error might normally be rejected as reflecting unacceptably strong modeling assumptions. In this case, however, the strong assumptions are not being made by the analyst but rather are guaranteed by the constructor of the test. Even in cases where the test was not constructed explicitly with IRT in mind, IRT models can fit well and provide strong information about measurement error. It would be foolish not to use these assumptions to the fullest extent, since they are available to us by construction.

### Estimation in IRT models

As with most statistical models, a variety of methods are used to estimate IRT models (see Sijtsma and Junker 2006). Here we treat only the most common maximum likelihood and Bayesian methods.

Since *θ_i_* is a random effect it is typical to assume *θ_i_* is iid Normal, with mean 0 and variance *τ*^2^ to be estimated. This leads to the marginal likelihood for *N* examinees and *J* items,

(6) $$L(a_{1},\ldots ,a_{J},b_{1},\ldots ,b_{J},c_{1},\ldots ,c_{J},\tau^{2})=\prod_{i=1}^{N}\int \prod_{j=1}^{J}P_{j}{\left(\theta_{i}\right)}^{x_{ij}}{\left(1-P_{j}(\theta_{i})\right)}^{1-x_{ij}}N(\theta_{j}|\mu ,\tau^{2}),$$

where *μ* = 0 is assumed, and all other parameters are to be estimated. In some situations the *μ* = 0 assumption and even the simple normality assumption may be relaxed, as in the next Section, for example.

The *a*, *b*, *c* and *τ*^2^ parameters may be estimated from equation 6 using direct maximum likelihood, an E-M algorithm, or Bayesian methods (after endowing all of the parameters with suitable prior distributions). In practice there is little difference between ML and Bayesian estimates for these models (Fox 2010). Because of this, and because of its convenience for constructing and estimating hybrid models, we focus on Bayesian estimation for the remainder of the paper.

Estimates of *θ_i_* in IRT models are typically fully Bayesian (that is, jointly estimated with the *a*, *b*, *c* and *τ*^2^ parameters) or some form of empirical Bayes estimates (that is, conditional on point estimates of the *a*, *b*, *c* and *τ*^2^ parameters). Estimates of *θ_i_* should be used in place of total score *X_i_* whenever possible, because they make more efficient use of cognitive testing data, and because their measurement error properties are well understood, as sketched below.

## Accounting for cognitive score measurement error

In order to use *θ_i_* in an analysis like that of equation (1), we must either (a) combine the IRT model with the regression model to estimate coefficients in (1) directly, or (b) provide estimates or imputations for each *θ_i_* that incorporate suitable measurement error into equation (1). We consider each method in turn.

### Joint modeling to account for cognitive score measurement error

Schofield (2008) sets out a mixed effects structural equations (MESE) model, which we employ here for the problem of adjusting our key regression (1) when we replace *θ* with a fallible test score.^c^ The latent cognitive variable is a random effect and the IRT and linear model parameters are all fixed effects, so this is a “mixed effects” model.

Mislevy (1991) shows, in the context of estimating subpopulation parameters for the National Assessment of Educational Progress (NAEP) and similar large-scale surveys, that incorporating latent variables from a measurement model into a regression analysis requires more elaborate random-effects distribution for *θ_i_* than shown in equation 6 above. Instead of assuming *μ* = 0 in the *n*(*θ*|*μ*, *τ*^2^) density shown there, we must assume that *μ* = *α*_0_ + *α*_1_*Z_i_* + *α*_2_*W_i_* (with *α*_0_, *α*_1_ and *α*_2_ to be estimated). The need for this more elaborate conditioning is also discussed by Schofield et al. (2012).

Thus, for equation (1) the MESE model takes the form

(7) $$y_{i}|Z_{i},W_{i},\theta_{i}\sim N(\beta_{0}+\beta_{1}\theta_{i}+\beta_{2}Z_{i}+\beta_{3}W_{i},\sigma^{2})$$

(8) $$x_{ij}|\theta_{i}\sim \mathrm{IRT}(x_{ij}|\theta_{i},\gamma_{j})$$

(9) $$\theta_{i}|Z_{i},W_{i}\sim N(\theta_{i}|\alpha_{0}+\alpha_{1}Z_{i}+\alpha_{2}W_{i},\tau^{2})$$

where *θ_i_*, *Z_i_*, *W_i_* and *y_i_* have the same roles as in (1) and *IRT*(*x_ij_*|*θ_i_*, *γ_j_*) is a suitable IRT model with parameters *γ_j_* for each item *j* (e.g., for the 3PL model, *γ_j_* ≡ (*a_j_*, *b_j_*, *c_j_*)). Equation (7) corresponds to equation (1), and is the regression of primary substantive interest.

Equation (7) is easily modified to accommodate logistic regression, or any other generalized linear model. In the case of logistic regression, *y_i_* becomes an indicator variable and equation (7) becomes

(10) $$\begin{array}{ll} y_{i} & \sim \text{Bernoulli}(p_{i})\\ \log\frac{p_{i}}{1-p_{i}} & =\beta_{0}+\beta_{1}\theta_{i}+\beta_{2}Z_{i}+\beta_{3}W_{i} \end{array}\}.$$

The MESE model solves some of the known identification problems often associated with errors-in-variables models. Typically, errors-in-variables models cannot be identified unless there is additional data in one of three areas: (1) replicate measures of *X_i_*, the observed test score, (2) distribution information on *θ_i_*, the unknown true score, or (3) distributional or moment restrictions on the error distribution (Stefanski 2000). By embedding the IRT model into the MESE model, we exploit the IRT model to provide further information about the measurement error of the cognitive test score.

The MESE model can be thought to have *J* measures of *X_i_* where *J* is the number of items on the test. For a test that is longer than one item, replicate measures (albeit crude measures) of *X_i_* are available. The IRT model uses these multiple measures of *X_i_* to estimate the Fisher Information *I*(*θ_i_*), which is then used to provide information about *SE*(*θ_i_*).

As for identification of the IRT model itself, the location and scale parameters of the latent distribution are confounded with the difficulty and discrimination parameters of the measurement model. In the 3-PL model, for example, the scales of *b_j_* and *θ_i_* are identified only up to an additive constant and the scales of *a_j_* and both *θ_i_* and *b_j_* are identified only up to a multiplicative constant. This indeterminacy of the IRT scales has ramifications for the regression coefficients; the choice of the latent scale of *θ_i_* affects the scale of *β̂*_1_. This choice does not affect statistical tests for the significance of *β̂*_1_, nor does it affect estimated coefficients for other covariates (Schofield 2008).

The indeterminacy of the IRT scales is easily dealt with, by fixing the scale of *θ* (e.g., *μ_θ_* = 0 and *σ_θ_* = 1.) Even when the item parameters are unknown and must also be estimated, there are well-known estimation methods (e.g, Bayesian MCMC, Patz and Junker 1999) to appropriately fix the scale of *θ* and the item parameters to ensure that the model is identified.^d^

As discussed below, in many empirical applications in economics analysts simply substitute a test score into regressions such as (7), thereby ignoring variation inherent in the measurement of cognitive ability. Exceptions include important recent work by James Heckman and co-authors: Hansen et al. (2004) and Cunha and Heckman (2008), for example, extend classical test-score models (Lord and Novick 1968) and the subsequent MIMIC approach (Joreskog and Goldberger 1975), which deals with measurement issues for posited latent variables when there are multiple observed indicators.^e^ These papers provide a template for the treatment of latent variables in applications for which multiple measures are available—usually a small number of measures. As we have noted, the MESE approach uses this same logic, but we are working with a case in which a typically larger number of replicate measures (generally “item responses”) are available, and those measures take a form that is appropriately handled by an embedded IRT model.

### Multiple imputation to account for cognitive score measurement error

Starting with the U.S. National Assessment of Educational Progress (NAEP) in the 1980’s (as reviewed by Mislevy et al. 1992), many large scale educational surveys release multiple *plausible values* (PVs)—known in the statistics literature as *multiple imputations* (Rubin 1987)—for each examinee’s proficiency, rather than a single proficiency estimate. PVs are draws from a posterior distribution of *θ_i_* for individual *i*, given that individual’s responses to items on a test and a set of background characteristics in a “conditioning model.” Typically, agencies release five PVs for each individual, and secondary analysts are instructed in the use of PVs for estimating statistics.

PVs solve three related problems for these agencies: (1) by law, certain government surveys such as NAEP are proscribed from releasing individual test scores to the general public, and PVs provide a potential way of protecting individual confidentiality; (2) many more test questions are used by these surveys than one examinee can respond to, and PVs provide for comparison on a common scale regardless of the difficulty of the questions asked; and (e:3pl) PVs are constructed to represent the uncertainty (measurement error) inherent in using a finite number of tasks or test questions to measure an unobservable latent construct such as literacy or math proficiency.

If *M* sets of PVs are made available by the survey agency, then a regression equation like (1) should be fitted *M* times, once with each set of PV’s. Results are then combined as recommended by Mislevy et al. (1992).^f^

When a well-constructed ensemble of PVs is used in accordance with their construction (e.g., Mislevy 1991 and 1993, and Li et al. 2009) in a secondary analysis, biases due to measurement error in cognitive score are negligible. Indeed, the posterior distribution from which plausible values are drawn is typically given by a model similar to that of equations (8) and (9) alone, with *Z* and *W* enlarged to include (proxies for) all possible regressors or contrasts that a secondary analyst might use.^g^ Except for the larger conditioning model for *θ*, correct use of PVs in (1) is in essence the same as estimating coefficients in (1) using MCMC methodology based on the MESE model.

There are three practical problems with PV methodology. First, there are only a few national and international surveys with the resources to produce PVs for secondary analysts. Second, PVs released by survey agencies involve *θ*s conditioned on many more variables than are needed in any particular secondary analysis, and can create additional problems (Schofield et al. 2012). Third, secondary analysts may be tempted to sidestep the correct procedure for using PVs as outlined by Mislevy (1991) and others. For example, in their study of international comparisons of wage inequality, Blau and Kahn (2005) treat the means and medians of individuals’ PVs in the 1994–96 International Adult Literacy Survey (IALS; Murray et al. 1997) as if *they* were the accurately measured levels of cognitive skills. von Davier et al. (2009) argue that this undermines the bias-correction built into PVs.

## Applications in labor economics

### Analyzing racial disparity in labor market outcomes

Several applications of equation (1) are found in labor economics. In this context the data is usually observational, from surveys such as the 1979 National Longitudinal Study of Youth (NLSY79; Zagorsky et al. 1997) or 1992 National Adult Literacy Survey (NALS; Kirsch et al. 2000), the outcome *y_i_* is a market outcome or individual choice (e.g., log wage, labor force participation, or educational attainment), *Z_i_* is an indicator of reference (*Z_i_* = 0) vs. focal (*Z_i_* = 1) group (e.g., white vs. black), *θ_i_* is a cognitive test score, and *W_i_* are any other relevant explanatory variables.

Neal and Johnson (1996) provide a landmark example. In that study the authors evaluate the role of cognitive skills acquired by youth (prior to entry in the labor market) on subsequent wage outcomes in U.S. labor markets. Using data from the NLSY79—which include individuals’ scores the Armed Forces Qualifying Test (AFQT)—the authors show that most of the black-white wage gap can be traced back to cognitive skills differentials that emerge at young ages.

Another example is Ritter and Taylor’s (2011) examination of racial differences in unemployment, using the same data and same basic approach as Neal and Johnson (1996). They find that black individuals have substantially higher levels of unemployment over their work careers than their white counterparts, and show further that much of this gap remains after accounting for racial differences in cognitive skills as measured by the AFQT.

The same basic structure appears in Lang and Manove’s (2011) test of a model in which young individuals’ educational attainment decisions are determined by their existing cognitive ability and by one’s race (owing to race-based differences in the way the education signal is perceived by employers). Using the NLSY79, the authors show that individuals with stronger cognitive skills (higher AFQT scores) are more likely to pursue higher education, and, consistent with the posited theory, they find that among individuals with similar cognitive skills, black men and women are more likely than their white counterparts to pursue higher education.

In each of the examples listed above, a cognitive test score is used as an explanatory variable, rather than treated as a latent construct. The hope presumably is that bias introduced is not too large. At the present time, items response data are not available for the AFQT for NLSY79 respondents, so it is not possible to assess empirically how problematic errors-in-variables bias might be in the specific applications listed above. Instead, in our analyses below we use data from similar sources, for which we do have item response data.

### Black-white wage differences in the U.S

To illustrate the two direct modeling approaches for dealing with the measurement error inherent in cognitive test scores—the MESE model and the use of PVs—we begin with an example that uses data from the NALS. These data include an individually-administered household survey of 24,944 adults aged 16 and over. The NALS is comprised of two sets of questions: standard demographic questions (race, gender, labor force behavior, marital status, education, etc.) and cognitive items that measure functional literacy in three domains: prose, document, and quantitative. The NALS was designed with 165 items to test the literacy skills of examinees, but each examinee was administered a representative sub-sample of approximately one-third of the full set of 165 items. Items not answered are treated as missing at random in the analyses below, as is common practice for designed missingness due to fractional designs and the like.

Our focus in our example is on racial differences in log wages. Table 1 provides some demographic characteristics of the NALS sample for blacks and whites. Three features merit attention. First, on average white men earn more than black men and white women earn more than black women. Second, on average black adults have relatively less education. Third, literacy skills, as measured by mean of plausible values, are relatively lower for black individuals than white individuals. NALS public-use data files from the National Center for Education Statistics (NCES) contain only basic data elements; in order to access individual cognitive item responses necessary for the IRT and MESE models, we also obtained restricted-use files from NCES.

We restrict our attention to just two subsets of the full NALS data set: *men*, married or single, aged 25–55, who work full time (work at least 35 hours for pay or profit during the week of their interview, either in one full-time job or in two or more part-time jobs), who report wages, and who answered at least one literacy item; and never married *women* who meet the same age, work and reporting criteria. The two groups are fitted separately because labor market outcomes might differ for men and women. Married women are excluded from our analyses because of the difficulty of establishing their work experience.^h^

Our interest is the comparison of estimated coefficients when we ignore measurement error in the literacy measure (i.e., the “unadjusted” case) and when we make appropriate “adjustments” using the MESE model, i.e., equations (7)–(9). The NALS data also contains *M* = 5 plausible values per content area and individual, constructed using the methods outlined above. This allows us to also compare estimates when we make appropriate use of PVs.

In all of the analyses reported here, *y_i_* is the log of self-reported weekly wage, *Z_i_* = 1 if the individual identifies as black (and *Z_i_* = 0 if white), and *W_i_* is a vector containing three covariates: “potential experience” (current age minus years of schooling minus 6) entered as a quartic, urban/rural status (a binary indicator), and census region (an unordered factor). For unadjusted analyses, *θ_i_* is replaced with an IRT-based ML estimate of total score on the entire 165 item pool.^i^ For adjusted analyses using the MESE model, *θ_i_* is merely the latent variable that links equations (8) and (9). For adjusted analyses using PVs, the model (1) is fitted five times, once for each set of PVs, and the results are combined using the using jackknife method recommended by Mislevy et al. (1992).

Both the unadjusted and adjusted models were estimated using Bayesian methods, in particular using an MCMC algorithm specified in WinBUGS. This was done to enhance comparability of estimates across models. Model fit for all models was compared using the DIC fit statistic from WinBUGS (Spiegelhalter et al. 2002). All parameters are estimated, except for item parameters in the IRT model.^j^

For the unadjusted model involving only equation (7), we used flat *N*(0, 10000) priors on each *β* coefficient, and a Unif(0, 1000) prior on *σ*^2^. Bayesian estimates with these priors are extremely similar to OLS estimates.^k^ For the full MESE model, we used the same priors on the *β*’s and *σ*^2^ in (7), and we fixed the item parameters to their NCES-estimated values in (8). In (9) we use an inverse-Gamma(1,1) prior on *τ*, and we assume flat *N*(0, 100) priors on each *α* coefficient. In order to further set the scale of *θ*, we mean-centered each of the covariates in (9). These priors on *θ_i_* allow for the possibility that blacks and whites, and people of different experience levels, census regions, and urban/rural status, have different distributions of proficiency.

We begin with a comparison of “unadjusted results” and MESE estimates. See Table 2. Columns (a) give baseline regressions in which we have no cognitive skill (i.e., literacy) measure. Columns (b) give results in which we add point estimates of literacy as a regressor. Finally, columns (c) give comparable estimates using the MESE model, equations (7)–(9), appropriately adjusting for the latent structure of the cognitive measure.

Standard arguments (Schofield 2008) suggest that if we ignore the errors-in-variables problem, we are likely to bias estimates of *β*_1_ toward zero, and more importantly for our purposes, bias estimates of *β*_2_ downward. With this in mind, consider first our estimates of *β*_1_. Coefficients reported in columns (b) and (c) are not directly comparable, since they depend on the scale of the cognitive measure. A better comparison can be made by examining the estimated effect of an increase in skills equal to one standard deviation (as measured using the white population). For men this is seen to be 0.190 under the unadjusted model (column (b)) and 0.218 under the adjusted model (column (c)). Results are similar for women. In short, we observe the attenuation bias in the expected direction in the (b) columns.

More importantly, we see that for both men and women, failure to account appropriately for the latent structure of cognitive ability leads to bias in estimates of the effect of race in our wage regression. As expected, estimates of *β*_2_ are biased downward in the unadjusted cases.

As discussed above, a defensible alternative approach to estimating equation (1) entails the appropriate use of plausible values (PVs), the multiple imputations of cognitive scores provided by some survey agencies in large scale surveys such as NALS. In Table 3 we compare wage-equation estimates from our MESE model, with two possible approaches for using plausible values (PVs). Columns (a) repeat our results for the MESE model from Table 2; columns (b) report results using the five sets of PVs provided by NCES with the NALS data set, combined using the procedure outlined in the Section entitled, Multiple imputation to account for cognitive score measurement error; and columns (c) report the result of using median PVs as a regressor in the wage equation (1). von Davier et al. (2009) provide formal arguments and Schofield (2008) provides informal arguments about potential biases for this sort of procedure–upward bias in the estimates of both *β*_1_ and *β*_2_.^l^

In Table 3, as in Table 2, estimates of return to skills (*β*_1_) are not directly comparable, because of scale dependence. However, estimates of the effect of a one SD increase in skills are very similar in columns (a) and (b), but somewhat inflated in columns (c), as expected given arguments in von Davier et al. (2009). Similarly, for both men and women, *β̂*_2_ is reasonably similar in columns (a) and (b), but appears biased upward in the (c) columns.

As discussed in the preceding Sections of our paper, either of the estimation procedures in columns (a) or (b) of Table 3 are defensible. The MESE model used in column (a) is designed to take full advantage of the direct model for measurement error that comes with NALS, and the PV method in column (b) duplicates this approach, using multiple imputations designed for secondary users. Numerical differences between columns (a) and (b) are small, and can be attributed to differences in the “conditioning model” expressed in equation (9): in our MESE model, only variables used in the wage equation were included in the conditioning, whereas for PVs, equation (9) is expanded to condition on (proxies for) all possible regressors and interactions that secondary analysts might use. For more details on differences one might expect to see with different conditioning models in (9), see Schofield et al. (2012).

The primary message from our empirical exercise is that the use of a “cognitive ability measure” as an error-free independent variable in a wage regression can lead to quite different inferences than a more defensible approach (MESE) that treats cognitive ability as a latent construct. For example, for men the estimated portion of the black-white log wage gap that is “unexplained” once we control for ability is −0.09 in our MESE model, which is more than one third smaller than the −0.14 estimate we get when we use the ML estimate of cognitive ability as a regressor. Our estimate also differs substantially from the −0.065 estimate that comes if we use the median PV of cognitive ability as a regressor.

It is important to note that our estimates rely on a *contemporaneous* measures of skills, which is the consequence of skills development when individuals are young, which could be shaped by disparate pre-market treatment, *and* skills development among individuals over time, which could be shaped in part by disparate treatment in the labor market. Our results are not directly comparable to those of Neal and Johnson (1996), for example. Although the regression framework is similar, conceptually their study is quite different: they use a measure of cognitive skills taken when individuals are quite young (still teenagers)—prior to their completion of school and entry into the labor market. Thus, their regression conceptually allows an assessment of the role of racial disparities of *pre-market* human capital development on eventual labor market outcomes. Our estimates might be of independent interest; for example Ferrer et al. (2006), Murnane et al. (1995), and Blau and Kahn (2005) all use contemporaneous measures of literacy skills in analyses of various sorts.

### The Black-white educational attainment gap in the U.S

Lang and Manove (2011) recently investigated the possibility that education is a generally more valuable signal of productivity for blacks that it is for whites. If so, their model predicts that young black individuals will invest more heavily in education than comparably-skilled whites. Evidence in support of this prediction comes from regressions that use data from the 1979 National Longitudinal Survey of Youth (NLSY79). In their regressions “educational attainment” is the dependent variable, and explanatory variables include a race indicator variable and a measure of cognitive ability (the AFQT). For most levels of the AFQT score, black men are found to have higher educational attainment than similarly skilled white men, and the same is true for women.

We estimate a similar regression to Lang and Manove (2011) using a different data source, the 1997 National Longitudinal Survey of Youth (NLSY97).^m^ This survey follows 8,894 youth born between 1980 and 1984. At the time of their first interview, individuals were aged 12–18. Since 1997, surveys have been conducted every year with data gathered on education attainment and enrollment, race, gender, and many other demographic items. Additionally, respondents have taken a standard skills assessment, the Peabody Individual Achievement Test-Revised mathematics assessment (PIAT; Markwardt 1998). We are therefore examining the determinants of the attainment of higher education for a more recent cohort than in Lang and Manove’s (2011) analysis.

The PIAT mathematics assessment contains 100 multiple choice items written to test the knowledge and application of mathematics concepts and facts, ranging from concrete problems like number recognition to more abstract problems like trigonometry. To save time, PIAT items are ordered from easiest to hardest and each individual is administered a customized set of items between those that are too easy (student would get them all correct) and those that are too hard (student would get them all incorrect). The raw PIAT score calculated from the individual’s item responses is, effectively, an estimate of the individual’s total score on all 100 items. This raw score is then converted into a standard PIAT score, normed by age to have mean 100 and standard deviation 15 in each age group; the usual NLSY public use data contain these standard PIAT scores. In addition, unlike the AFQT for which item responses are not available for either the NLSY79 or NLSY97, item response data have recently been made available for the PIAT assessment.^n^ Although an IRT model is not provided for the PIAT, we show below that a suitable IRT model fits the data well and provides a good direct model for measurement error.

Table 4 provides some demographic characteristics of the NLSY97 sample for the 2006 wave. A few features of the data are worth noting. First, individuals are still young enough that many are likely to attain additional education in coming years. Still, they are old enough that virtually no one is still in high school. A much higher proportion of blacks than whites in this sample have failed to complete high school, and a much higher fraction of whites than blacks have some post-secondary education. Second, on average, blacks have lower PIAT mathematics standard scores than whites. The average standard score is two-thirds of a standard deviation lower for blacks than for whites.

We proceed to analyze the role of race and measured cognitive ability on educational attainment. Our outcome variable of interest is *y_i_* = 1 if individual *i* has enrolled in a four-year post-secondary institution and 0 otherwise (as of 2006), and *y_i_* = 0 otherwise. Our basic model is the logistic regression model (10), where *p_i_* = *P*(*y_i_* = 1). As before we take *Z_i_* = 1 if the individual reports as black (*Z_i_* = 0 otherwise), and we include in *W_i_* covariates for age, urban/rural status (as a binary indicator), and census region (as an unordered factor). For a cognitive measure we consider both the standard PIAT score from the first (1997) NLSY data round, and a latent *θ_i_* provided by an IRT fit to the item-level data on which the standard PIAT scores were based. Our investigation is very much in the spirit of the Lang-Manove work; we are interested in the impact of race on the enrollment in a four-year institution conditional on the skill levels that young people hold prior to enrollment.

We compare estimates from the logistic regression (10) using standard PIAT scores, unadjusted for measurement error, with the logistic MESE model comprising equations (8), (9) and (10). Once again, all models were fitted using the Bayesian methodology entailed in the WinBUGS software.

For the basic logistic regression model (10), we used very flat *N*(0, 100) priors for the *β* parameters. Bayesian estimates using these priors are very similar to standard ML estimates of the same logistic regression model.^o^ For the full logistic MESE model, we used the same priors on the *β’*s in 10, and for *τ* in 9 we again used an inverse-Gamma(1, 1) prior and we assume flat *N*(0, 100) priors on each *α* coefficient. In order to set the scale of *θ*, we mean-centered each of the covariates in (9). In line with our discussion above, the prior on *θ_i_* is conditioned on race, age, census region, and urban/rural status, thereby allowing for the possibility that there are differences in distributions of proficiency for blacks and whites of different age groups, census regions, and urban/rural status. We also estimated item parameters in (8), since we do not have them from the test publishers. For each discrimination parameter *a_j_* we used a Γ(1, 1) prior, and for each guessing parameter *c_j_* a *Unif* (0, 1) prior. Finally, for each difficulty parameter *b_j_* we used a normal prior with a standard deviation of 0.1 and a mean dependent on the item number ranging from −4 to 4. The priors on the difficulty parameter reflect the PIAT’s structure that item 1 is supposed to be easier than item 2 which should be easier than item 3 and so on.

Before proceeding with the full analysis we did a preliminary fit of our IRT model to the PIAT data, because the PIAT test was not (to our knowledge) originally constructed using the IRT model. Regardless, IRT models can fit well and can still provide us with information about the measurement error. We use the “outfit” mean square statistic *T_j_*(*x*|*θ*, *γ*) (Johnson et al. 1999) to diagnose possible misfit of any particular item,

(11) $$T_{j}(x|\theta ,\gamma )=\sum_{i=1}^{N}\frac{x_{ij}-E_{ij}}{NW_{ij}},$$

where *x_ij_* is respondent *i*’s response to question *j*, *E_ij_* and *W_ij_* are the expected value and variance respectively of *x_ij_* conditional on the item parameters and *θ*. Because the outfit statistic is conditional on the item parameters and *θ*, we calculate posterior predictive *p*-values (Gelman et al. 1996). Posterior predictive *p*-values allow us to average over the uncertainty in *θ* and *γ* using *M* simulated replicated datasets (x^*^) from the predictive distribution of the data. We then estimate the posterior predictive *p*-value as

(12) $$p\approx \frac{\#s:T_{j}(x|\theta_{s},\gamma_{s})<T_{j}(x_{s}^{*}|\theta_{s},\gamma_{s})}{M};s=1,\ldots ,M$$

If the value of the posterior predictive *p*-value is small, there is reason to be concerned about the fit of our model for that item.

For the 100 items on the PIAT (with an *M* = 1000, our posterior predictive *p*-values range in value from 0.182 to 0.674. Therefore, the IRT model fits quite well and provides a good direct model for PIAT measurement error.

Since NLSY did not produce plausible values for PIAT scores, our analysis does not include a comparison with PV methodology.

In Table 5 we provide estimates for three versions of our regressions, separately for men and women. Columns (a) and (b) report results using the logistic regression model (10), without any cognitive measure (columns (a)), and using the standard PIAT score as a cognitive measure, unadjusted for measurement error (columns (b)). The (c) columns report the result of the logistic MESE model.

As in Table 2, the results in Table 5 reflect well-known attenuation bias in assessing the impact of cognitive ability on the outcome of interest, as is seen by comparing the estimated effects in columns (b) with columns (c) of a one standard deviation changes in the skills measure on college enrollment.

More important are our inferences regarding the role of race. When we treat the PIAT score as a regressor, in the (b) columns, we infer the black men are substantially less likely than similarly-skilled white counterparts to enroll in college. We infer that comparably-skilled black and white women are equally likely to enroll in college.

Inferences are quite different when we use the MESE model. Results reported in the (c) columns suggest that black men are in fact as likely as their similarly-skilled white counterparts to enroll in college, and that black women are *more* likely to enroll than comparable white women.^p^

It is worth noting that on the basis of the regressions that follow standard practice (reported in the (b) columns) we would have rejected the hypothesis that blacks get more education that whites with similar levels of cognitive aptitude. The MESE approach, in contrast, is reasonably consistent with the Lang-Manove hypothesis, particularly for women. Again, recall that individuals in our sample were quite young (average age 24). As data become available with successive waves of the NLSY97, it will become possible to shed additional light on the racial differences in completed education, and the role of cognitive skills (developed among young students) in the educational-attainment decision.

## Conclusions

Many analyses in labor economics, and in the social sciences more generally, entail estimation of regressions in which “cognitive ability” *θ_i_* appears as an explanatory variable. It this paper we have investigated problems that arise with the standard practice of simply using a test score as a regressor in this context.

Our central point is that any candidate point estimate of *θ_i_* entails measurement error. When *θ_i_* is obtained from a well-constructed cognitive assessment using standard modern technology, a direct model for measurement error is usually available in the form of an item response theory (IRT) model. Indeed, many cognitive tests are constructed specifically so that the data is well-fit by an IRT model. The existence of IRT as a direct model for measurement error obviates the need for such remedies as nonparametric bounds and instrumental variable methods. Indeed, because the cognitive assessment was constructed to fit this model, answers obtained using the IRT model have more authority than answers using other methods, regardless of raw comparisons of effect size estimates, statistical significance, etc.

In this paper we have discussed two essentially equivalent approaches to incorporating the IRT model directly into regression analyses using a cognitive measure as an independent variable: directly fitting the mixed-effects structural equations (MESE) model of Schofield (2008), and, when available, the use of multiple imputations of cognitive skill measures known as plausible values (PVs; Mislevy et al. 1992). With two illustrative analyses, a linear and a nonlinear regression, we show that failing to account properly for measurement error produces predictable biases, which can lead to serious misunderstandings.

Our work leads us to a final observation. Analysts who use secondary data are obviously at the mercy of the teams that collect and release data; analysts can only use data that are made available. In cases where researchers want to estimate models in which cognitive ability (or other latent constructs) are used as an explanatory variable, it is essential that those data include item response data or, at a minimum, well-constructed plausible values. It is important that the research community communicate the value of such data to agencies who collect and disseminate data.

## Figures and Tables

**Table 1:** Sample characteristics, 1992 National Adult Literacy Survey (NALS)

	Black men	Black women	White men	White women
N	1665	2807	7449	9404
Average Age	39.4	39.4	40.5	42.4
Marital Status				
Proportion never Married	0.39	0.38	0.28	0.19
Education				
Proportion Still in HS	0.06	0.04	0.04	0.03
Proportion *<* HS	0.29	0.29	0.13	0.14
Proportion HS	0.29	0.29	0.27	0.30
Proportion *<* College	0.25	0.29	0.30	0.33
Proportion College +	0.11	0.09	0.26	0.21
Literacy Skills				
Mean *Plausible Value*	−0.66	−0.50	0.48	0.50
St. Dev. *Plausible Value*	1.14	1.18	1.03	0.96
Earnings of Full-Time Workers				
Average Weekly Wage	452.3	397.5	674.6	440.9

Notes: Authors’ calculations, National Adult Literacy Survey.

**Table 2:** Log wage regressions

	Men			Never married women		
Model	Unadj.	Unadj.	MESE	Unadj.	Unadj.	MESE
		
Skill control	No skill control	MLE of Lit score	Literacy skill	No skill control	MLE of Lit score	Literacy skill
	(a)	(b)	(c)	(a)	(b)	(c)
Lit. Skills: (*β̂*_1_)						
Unadjusted		0.151 (0.008)			0.153 (0.020)	
Adjusted (MESE)			0.191 (0.010)			0.185 (0.025)
Effect of a one SD		0.190	0.218		0.186	0.210
Change in Skills						
Race (*β̂*_2_)	−0.366 (0.033)	−0.144 (0.033)	−0.094 (0.033)	−0.233 (0.055)	−0.049 (0.057)	−0.012 (0.060)

DIC	5904	5577	103839	1191	1114	20905
N	3267	3267	3267	640	640	640

Notes: Data are from the 1992 NALS, restricted to individuals aged 25–55 who work fulltime, reported wages, and who answered at least one literacy item. Unadjusted regressions employ the wage equation (1) with either no cognitive measure (column a) or a measure unadjusted for measurement error (column b). Column (c) provides estimates from the MESE model, equations (7)–(9), adjusting for measurement error in the cognitive measure. All regressions also control for potential experience entered as a quartic, census region (entered as dummy variables), and urban setting (entered as a dummy variable).

**Table 3:** Plausible-values adjustments for log wage regressions

	Men			Never married women		
Model	MESE	PVs	Unadj.	MESE	PVs	Unadj.
		
Skill control	Literacy skill	All PVs	Median PV	Literacy skill	All PVs	Median PV
	(a)	(b)	(c)	(a)	(b)	(c)
Lit. Skills (*β̂*_1_):						
MESE	0.191 (0.010)			0.185 (0.025)		
All PVs		0.221 (0.015)			0.220 (0.033)	
Median PV			0.276 (0.012)			0.276 (0.031)
Effect of a one SD	0.218	0.221	0.251	0.210	0.220	0.259
Change in Skills						
Race (*β̂*_2_)	−0.094 (0.033)	−0.121 (0.041)	−0.065 (0.033)	−0.012 (0.061)	−0.031 (0.062)	0.022 (0.059)
DIC	103839	5492	5462	20905	1127	1114

N	3267	3267	3267	640	640	640

Notes: Data are from the 1992 NALS, restricted to individuals aged 25–55 who work fulltime, reported wages, and who answered at least one literacy item. MESE model estimates (column a) are from Table 2. “All PV’s” estimates (column b) employ the recommended procedure (Mislevy et al. 1992) for combining regression results for multiple imputations. “Unadjusted Median PV” estimates (column c) employ the median PV in the wage equation (1), with no adjustment for measurement error. All regressions also control for potential experience entered as a quartic, census region (entered as dummy variables), and urban setting (entered as a dummy variable).

**Table 4:** Sample characteristics, National Longitudinal Study of Youth 1997 (NLSY97)

	Black men	Black women	White men	White women
N	1169	1165	2286	2127
Avg. Age	24.4	24.5	24.3	24.3
Education				
Proportion Still in HS	0.003	0.007	0.002	0
Proportion < HS	0.16	0.11	0.07	0.06
Proportion HS/GED	0.32	0.25	0.26	0.19
Proportion Some College	0.30	0.43	0.34	0.35
Proportion College +	0.05	0.11	0.16	0.23
Math Skills				
Mean Std PIAT Score	88.06	88.67	98.99	98.39
St. Dev Std PIAT Score	14.57	14.51	14.04	13.55

Notes: Authors’ calculations, National Longitudinal Survey of Youth 1997.

**Table 5:** Four year college enrollment, logistic regressions

	Men			Women		
Model	Logistic	Logistic	MESE	Logistic	Logistic	MESE
		
Skill control	No skill control	Standard PIAT score	PIAT	No skill control	Standard PIAT score	PIAT
	(a)	(b)	(c)	(a)	(b)	(c)
Lit. Skills (*β̂*_1_):						
Std. PIAT Score		0.058 (0.004)			0.052 (0.004)	
PIAT MESE			0.528 (0.050)			0.509 (0.048)
Effect of a one SD		0.814	0.950		0.704	0.817
Change in Skills						
Race (*β̂*_2_)	−0.768 (0.105)	−0.239 (0.120)	0.022 (0.128)	−0.495 (0.106)	0.005 (0.121)	0.258 (0.129)
DIC	2677	2424	63066	2500	2323	58076

N	2035	2035	2035	1853	1853	1853

Notes: National Longitudinal Survey of Youth 1997 for waves through 2006. All regressions control also for age, census region (an unordered factor), and urban/rural area (a binary indicator).

## Footnotes

a Examples for standardized tests used in regression analysis include the Armed Services Vocational Aptitude Battery (Department of Defense 1984), the Peabody Individual Achievement Test (Markwardt 1998), the National Assessment of Education Progress (Allen et al. 1999), and the National Adult Literacy Survey (Kirsch et al. 2000).

b Thus, black men earn (roughly) between 7% less than and 126% more than white men, and bounds for the estimated black-white gap for women are similarly large.

c The model is a form of “structural equations model” (SEM), as discussed, e.g., in Bollen (2002), and Fox and Glas (2003) in which the measurement model is an IRT model and the “structural model” is a normal linear model.

d Below we use Bayesian estimation. After supplying prior distributions for parameters it is straightforward to specify an MCMC algorithm using the WinBUGS software (Spiegelhalter et al. 2000).

e Additional contributions include Heckman et al. (2006) and Cunha et al. (2010). Importantly, this work deals with issues that arise in the evaluation of both cognitive and non-cognitive skills.

f Standard errors estimates of regression coefficients incorporate both model-based uncertainty and Monte-Carlo uncertainty; see Mislevy et al. (1992) for a complete overview.

g This remarkable fact follows directly from the work of Mislevy (1991) for example.

h Neal (2004) provides a discussion of the difficulties in assessing labor market effects of race for women. In our analyses, we control for “potential experience,” using essentially number of years past school, but for married women this is likely to be a poor measure of participation in the labor market outside the home. Moreover, the extent of the problem with this measure likely differs by race.

i This rather indirect calculation is needed because different respondents saw different subsets of the 165 item pool, and the subsets differ in difficulty. It is, essentially, an IRT-based *equating* (Kolen and Brennan 2004) of the different subsets of items seen by each respondent.

j Item parameter estimates obtained by NCES are provided in the NALS data set, and are based on such a large sample that their SE’s are essentially zero.

k For example, the unadjusted OLS estimate for the race coefficient in the no skills control regression for men is −0.365 and for women it is −0.236. Standard error estimates for both of these OLS estimates are the same as the Bayesian estimates.

l We provide this last set of results because this approach has been used by previous researchers.

m Like Lang and Manove, we are interested in the effect of race on the decision to attend college. However, our primary goal here is to compare results from ordinary regression to the MESE model, so we use a simpler specification; we do not enter the aptitude measure as a quadratic nor do we interact it with the race indicator variable.

n We are grateful to Dan Black for his role in making the data available.

o For example, the unadjusted ML estimate for the race coefficient in the no skills control logistic regression for men is −0.767 and for women it is −0.494. Standard error estimates for both of these ML estimates are the same as the Bayesian estimates.

p We are using a non-linear model here, so arguments about the bias that results when ignoring measurement error in linear models is not directly applicable. Nonetheless the bias is just as predicted by those simple arguments.
